# Dried blood spot sampling for hepatitis B virus quantification, sequencing and mutation detection

**DOI:** 10.1038/s41598-022-05264-1

**Published:** 2022-01-31

**Authors:** Cristianne Sousa Bezerra, Moyra Machado Portilho, Jakeline Ribeiro Barbosa, Carolina Pimentel de Azevedo, Ana Carolina da Fonseca Mendonça, José Napoleão Monte da Cruz, Cristiane Cunha Frota, Bárbara Vieira do Lago, Lívia Melo Villar

**Affiliations:** 1 Laboratorio de Hepatites Virais, Instituto Oswaldo Cruz, Helio and Peggy Pereira Pavillion - Ground Floor - Room B09, Fundação Oswaldo Cruz, FIOCRUZ, Av. Brasil, 4365 – Manguinhos, Rio de Janeiro, Rio de Janeiro 210360-040 Brazil; 2 Departamento de Educação, Instituto Federal de Educação, Ciência E Tecnologia Do Ceará, Fortaleza, Ceará Brazil; 3 Instituto de Pesquisas Gonçalo Moniz, FIOCRUZ, Salvador, Bahia Brazil; 4 Laboratório Central Do Estado Do Ceará, Fortaleza, Ceará Brazil; 5 Departamento de Patologia E Medicina Legal, Faculdade de Medicina, Universidade Federal Do Ceará, Fortaleza, Ceará Brazil

**Keywords:** Biotechnology, Microbiology, Molecular biology, Diseases, Health care

## Abstract

Hepatitis B virus (HBV) diagnosis is performed on serum samples, but the access to this diagnosis is difficult in low-income regions. The use of dried blood spot (DBS) samples does not require special structure for collection, storage or transport. This study evaluates the use of DBS for detection, quantification and sequencing of HBV DNA using *in-house* techniques. Two study groups were included: 92 HBsAg + individuals and 49 negative controls. Serum and DBS samples were submitted to quantitative and qualitative *in-house* PCR for *S/pol* genes, sequencing and phylogenetic analyses. Total of 84 serum samples were successfully amplified. Of them, 63 paired DBS were also positive in qualitative PCR. Qualitative PCR in DBS presented a sensitivity of 75% and specificity of 100% (Kappa = 0.689). Quantitative PCR in DBS presented a detection limit of 852.5 copies/mL (250 IU/mL), sensitivity of 77.63% and specificity of 100% (Kappa = 0.731). A total of 63 serum samples and 36 DBS samples were submitted to sequencing, revealing the circulation of genotypes A (65.08%), D (4.8%), E (3.2%) and F (27%) with 100% of correspondence between serum and DBS. All sequenced samples displayed polymorphisms in HBsAg gene. An HIV-coinfected patient presented the rtM204V/I-rtL180M double resistance mutation in serum and DBS. In conclusion, DBS is an alternative to detect, quantify and characterize HBV DNA, being a possibility of increasing diagnosis in low-income settings, closing gaps in HBV control.

## Introduction

The World Health Organization (WHO) has estimated that 257 million people have been chronically infected by the hepatitis B virus (HBV) worldwide. Because of this, WHO has suggested increasing the access to diagnosis as a key to reduce the number of new infections, thus preventing millions of deaths^1^. Once chronic infection can progress to more aggressive conditions, such as cirrhosis and hepatocellular carcinoma (HCC), early diagnosis and treatment are needed to reduce mortality, prevent HBV long-term complications and to avoid viral transmission^1–3^.

Molecular tests, as HBV quantification and sequencing, are useful for HBV diagnosis. The start and outcome of antiviral therapy are assessed by quantifying HBV DNA in serum or plasma. Molecular tests are also important for genotyping, monitoring of drug resistant and vaccine-escape strains, noncompliance of antiviral therapy and other possibilities^4–8^. However, commercial assays are usually expensive and consequently less accessible in low-income settings^9,10^. Furthermore, the use of serum samples can be an obstacle for molecular studies in hard-to-reach populations such as elder people, children, drug users and people living in remote places^11^. In all these cases, dried blood spot (DBS) samples may be an easy, economic and less-invasive alternative.

Studies have shown that HBV DNA can be detected and quantified in DBS samples^11–13^. The use of DBS has some advantages: the small quantity of blood can be provided by a finger-prick; DBS is easy to collect and it requires minimal training; this sample is stable at room temperature which makes it easier to transport from remote places to a reference laboratory and even allows sending the sample by mail^13–15^.

Thus, the advent of a quantitative *in-house* real time PCR that can be adapted for DBS samples could improve the access to HBV molecular diagnosis in areas of limited infrastructure^16,17^. Moreover, the use of DBS in molecular epidemiology studies, to better understand the role of genotypes and viral mutations in the course of hepatitis B is crucial to promote ways to access HBV diversity in settings where the gold standard methods are not available.

Once most HBV chronically infected individuals live in remote regions, with limited laboratory infrastructure to perform the virus diagnosis, the access to economical and accurate diagnostic methods is an essential step for the eradication of HBV. At our knowledge, scarce studies have tracked HBV genotypes, resistance and vaccine-escape mutations in DBS samples and most of them did not evaluate the reliability of DBS compared to their paired serum samples^12,18–21^.

The present study aimed to evaluate the usefulness of DBS for HBV quantification and sequencing, using *in-house* techniques.

## Results

### Characteristics of participants

A total of 141 individuals were enrolled in this study. Group 1 was composed by 92 HBV infected individuals, 53 (57.6%) were men; mean age was 44.26 ± 14.97 years. Group 2 had 49 healthy individuals, 35 (71.4%) were women; mean age was 37.12 ± 11.27 years.

Eighteen patients (19.6%) related previous antiviral treatment (lamivudine, entecavir or tenofovir). According to HBV risk behaviours, both groups reported frequently going to the manicure and sharing razors. However, 39/49 (79.6%) people from group 2 mentioned using their own pliers during the procedure. Another point is the use of condoms. Almost 40% of individuals from group I reported never using a condom during sexual intercourse in contrast to 12.24% from group 2 (p = 0.33). Regarding coinfections, 6 patients from group I presented HBV-HIV coinfection.

From group 1, 24 (26.1%) individuals were HBeAg positive, and 69 (75%) were anti-HBe positive. No patient was positive for HCV. Biochemical and serological profiles of the subjects are shown in Table 1.

**Table 1:** Biochemical, HBV, HCV and HIV serological markers among HBV infected individuals (n = 92).

Variable	Value
HBsAg Positive, N (%)	92 (100)
Anti-HBs Positive, N (%)	1 (1.09)
Anti-HBc* Positive, N (%)	88 (96.7)
HBeAg* Positive, N (%)	24 (27.59)
Anti-HBe* Positive, N (%)	69 (76.7)
Anti-HCV Positive, N (%)	0 (0)
Anti-HIV* Positive, N (%)	6 (6.6)
Indirect bilirubin, mean (± SD)	0.08 mg/dL (± 1.15)
Direct bilirubin, mean (± SD)	0.2 mg/dL (± 1.69)
Total bilirubin, mean (± SD)	0.31 mg/dL (± 0.78)
ALT, mean (± SD)	14 (± 238.74)
AST, mean (± SD)	22.5 (± 255.29)
Gama GT, mean (± SD)	25 (± 129.6)
Alkaline phosphatase, mean (± SD)	56 (± 51.91)

*SD* standard deviation.

*Totals are not from 92 subjects due to missing values.

### Analytical sensitivity: linear dynamic range and detection limit

A serial dilution panel was used to evaluate the linear dynamic range and detection limit of DBS samples. The lowest HBV dilution detected in qPCR was 3 log10 (3,410 copies/mL or 1,000 IU/mL). Then, this sample was twofold diluted, from 1/2 to 1/64, and these dilutions were subjected to another run of the same qPCR in duplicate. HBV DNA was detected until the dilution of 1/4, resulting in an estimated detection limit of 852.5 copies of HBV/mL or 250 IU/mL (Table 2).

**Table 2:** Mean CT and estimated viral log values determined by qPCR test according to the dilution panel.

Viral load (copies^mL-1^)	Estimated viral load (log copies/mL) Mean ± SD	CT value Mean ± SD
2 × 10^7^	5.89E + 05 (± 8.06E+04)	20.43 (± 0.11)
2 × 10^6^	5.78E + 04 (± 1.31E+04)	24.14 (± 0.05)
2 × 10^5^	8.12E + 03 (± 3.37E+03)	26.99 (± 0.18)
2 × 10^4^	7.74E + 02 (± 1.41E+01)	30.60 (± 0.79)
2 × 10^3^	1.64E + 02 (± 2.12E+00)	34.57 (± 0.59)
2 × 10^2^	ND	ND
2 × 10^1^	ND	ND
2 × 10^0^	ND	ND
(2 × 10^3^) × 1/2	53.85 (± 8.41)	34.24 (± 0.24)
(2 × 10^3^) × 1/4	27.9 (± 0.01)	35.23 (± 0.01)
(2 × 10^3^) × 1/8	ND	ND
(2 × 10^3^) × 1/16	ND	ND
(2 × 10^3^) × 1/32	ND	ND
(2 × 10^3^) × 1/64	ND	ND

The tests were conducted in duplicate to give viral load.

*SD* standard deviation, *ND* not detected.

* n = 2.

To assess the precision of qPCR, a DBS sample with HBV viral load of 4 log IU/mL (~ 4 log copies/mL) was quantified 20 times in the same run and the coefficient of variation (CV) was 0.73.

### Reproducibility and repeatability

To assess the reproducibility of the qPCR in DBS samples, high and low positive controls (HPC and LPC) were tested by three operators in three different days, twice a day, using the same reagents. Results are presented in Table 3. For HPC, a CT variation from 16.62 to 20.61 (mean of 17.56 ± 0.93) was observed, while for the LPC the variation was from 31.03 to 36.54 (mean of 33.65 ± 1.5), presenting CV of 0.05 and 0.04, respectively. There was no statistical difference between the mean CT values for both HPC and LPC demonstrating good reproducibility of the method.

**Table 3:** Repeatability and reproducibility analysis of HPC and LPC according to estimated viral load and CT obtained from qPCR in DBS samples.

Variable	Repeatability		Reproducibility	
	Mean ± SD (CV) Estimated viral load (log copies/mL)	Mean ± SD (CV) CT Value	Mean ± SD (CV) Estimated viral load (log copies/mL	Mean ± SD (CV) CT Value
HPC (10^10^)	4.40E + 06 ± 1.08E + 06 (0)	17.56 ± 0.93 (0.05)	4.39E + 06 ± 2.55E + 05 (0)	16.94 ± 0.09 (0.01)
LPC (10^4^)	75.53 ± 54.6 (0.72)	33.65 ± 1.5 (0.04)	92.51 ± 32.44 (0.35)	32.73 ± 0.48 (0.01)

*HPC* high positive control, *LPC* low positive control, *SD* standard deviation, *CV* coefficient of variation.

Regarding repeatability, viral loads of HPC in the same day conducted by the same operator were similar, while for LPC, variation of viral load was noted in the same reaction and in different days.

### Evaluation of qPCR using serum and DBS samples

Initially, serum samples from group 1 (n = 92) were submitted to HBV quantification by a commercial method, presenting median viral load of 2,645 IU/mL (333 to 1 × 10^9^), which corresponds to 10,698.10 copies/mL (1135.53 to 3.41 × 10^9^). HBV DNA was successfully detected by qPCR in 76 serum samples from group 1 (76/92 = 82.61%), presenting a median viral load of 534 copies/mL (2.05 to 3.84 × 10^10^). The 76 serum samples that were positive in qPCR had their paired DBS samples submitted to that same protocol, resulting in 63 DBS samples successfully detected and quantified (median of 41.3 copies/mL; 0.148–4.93 × 10^7^). These results demonstrated a sensitivity of 77.63% of qPCR using DBS when compared to serum, with agreement value of 86.40% and Kappa value above 0.7 (Table 4). Group 2 serum and DBS samples were negative in both commercial and qPCR. Positive predictive value (PPV) was 100% (95% CI 94.26–100) and negative predictive value (NPV) was 70% (95% CI: 57.87–80.37).

**Table 4:** Comparison between qualitative and quantitative (qPCR) HBV DNA detection of in serum and DBS samples.

Variable analyzed	Comparison	
	Qualitative PCR (serum) vs*.* qualitative PCR (DBS)	qPCR (serum) vs. qPCR (DBS)
Total (N)	133	125
Sensitivity% (CI 95%)	75 (64.36–83.81)	77.63 (66.62–86.41)
Specificity% (CI 95%)	100 (92.68–100)	100 (92.75–100)
True positive	63	59
True negative	49	49
False positive	0	0
False negative	21	17
PPV*% (CI 95%)	100 (94.26–100)	100 (93.56–100)
NPV**% (CI95%)	70 (57.87–80.37)	74.24 (65.47–81.42)
Kappa	0.689	0.731

*CI* confidence interval.

*Predictive positive value; **Predictive negative value.

**Figure 1:**
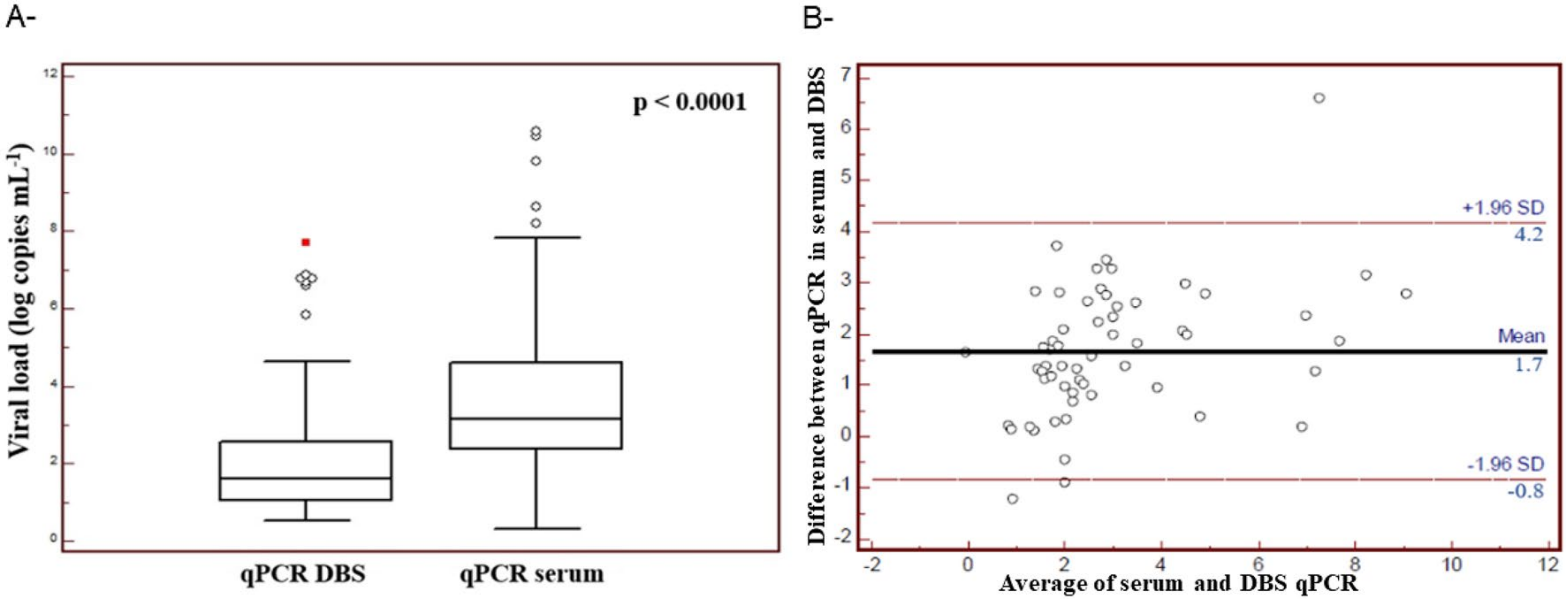
Quantitative analyses of HBV viral loads (log copies/mL) in serum and DBS samples (qPCR). (**A**) Box-Plot of HBV DNA viral load (log copies/mL) for paired DBS and serum using qPCR. The outliers are represented as the black circles. P < 0.0001 (Mann–Whitney Rank Sum Test). (**B**) Bland–Altman Plots resulting from the pairwise comparison of HBV viral load in serum and DBS samples in qPCR. The mean difference (black line at center) is presented as a function of the average viral load of the assays performed in two different samples. Upper and lower limits of agreement (upper and lower pink lines, respectively) defined by mean HBV viral load difference ± 1.96 Standard Deviation (SD).

When HBV viral load was compared between serum and DBS, significantly higher viral load was observed among serum samples, in comparison to DBS (3.03 vs. 1.62 log copies/mL). However, qPCR results for serum and DBS showed good correlation, as demonstrated by the coefficient of Pearson (r = 0.844; 95% IC: 0.747–0.905, p < 0.0001). Figure 1A shows the comparison of log copies/mL values between DBS and serum samples in qPCR. In this case, the qPCR in serum had a median equal to 3.03 while the qPCR in DBS had a result equal to 1.62.

Finally, the pairwise comparison of HBV viral load between qPCR in serum and DBS was performed using a Bland–Altman Plot (Fig. 1B). A mean point of 1.66 log copies/mL was estimated, with a standard deviation of 1.96 log copies/mL. The upper limit of agreement was 4.15 (95% CI 3.58–4.73) and the lower limit was − 0.83 (95% CI − 1.40 to 0.26). Three samples were outside the limits of agreement, with an estimated value of − 0.92; − 1.23 and 6.6; which represent 5.08% of the samples, an index slightly above the tolerated limit (5%). Within the region of agreement, there were differences between the measurements obtained by the two samples.

HBV DNA detection in DBS samples by qPCR was also evaluated according to serological and biochemical markers (Table 5). Median viral load detected in commercial test and serological markers as HBsAg (DO/CO values), HBeAg and anti-HBe were statistically related to HBV DNA quantification in DBS (< 0.0001).

**Table 5:** HBV DNA quantitative detection in DBS samples by qPCR correlated to laboratory variables.

Variable	qPCR DBS		
	HBV-DNA+	HBV-DNA -	p-value
qPCR serum^#^ (log copies/mL) Median (interval)	3.03 (0.31–10.58)	1.16 (0.39–2.12)	0.99
OD/CO* HBsAg Median (interval)	49.09 (20.00–142.86)	27.74 (17.42–142.86)	0.01^**+**^
ALT (U/L) Median (interval)	14.00 (0.00–678.00)	13.00 (2.00–44.00)	0.32^**+**^
AST (U/L) Median (interval)	21.00 (0.00–337.00)	22.00 (4.00–125.00)	0.86^**+**^
**HBeAg**			
Positive	21	30	< 0.0001^¶^
Negative	40	1	
**Anti-HBe**			
Positive	39	3	< 0.0001^¶^
Negative	22	28	
**Anti-HIV**			
Positive	4	2	1.000^¶^
Negative	57	29	

*OD/CO = optical density/cut-off; ^+^ Mann–Whitney test; #HBV-DNA quantified in serum by qPCR; ^¶^ Fisher test.

### HBV-DNA qualitative detection and nucleotide sequencing

From all 92 HBV positive samples (group 1) submitted to qualitative PCR for *S/Pol* genes, 84 serum samples were successfully amplified. Of them, 63 paired DBS were also positive in qualitative PCR, giving the reaction a sensitivity of 75% (95% CI 64.36–83.81). Since all 49 samples from group 2 (paired serum and DBS) tested negative in qualitative PCR, the specificity of the reaction was 100% (95% CI 92.68–100) (Table 4).

**Figure 2:**
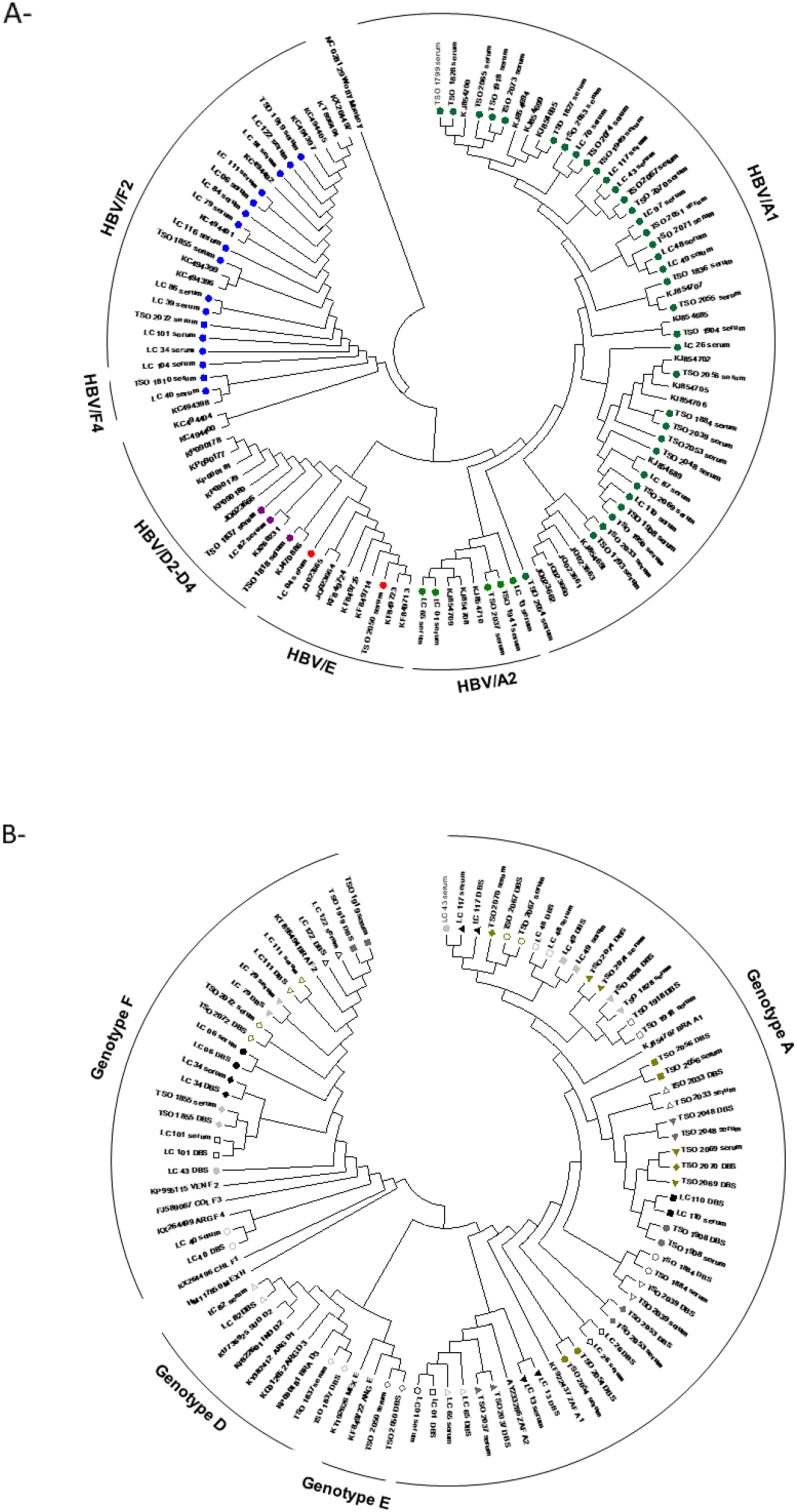
Phylogenetic analysis based on HBV S/Pol sequences performed by using the maximum likelihood method. (**A**) Phylogenetic tree composed by 108 HBV isolates, including 58 serum samples sequenced in this study. Green dots: HBV/A sequences; blue dots: HBV/F sequences; purple dots: HBV/D sequences; red dots: HBV/E sequences. (**B**) Phylogenetic tree composed by 90 sequences, including 36 paired serum and DBS sequences. Serum and DBS pairs are identified by the same graphical markers.

HBV-DNA was successfully sequenced in all 63 serum samples (100%) and in the respective 36 (57.14%) paired DBS samples (Fig. 2, Table 6). Genetic distance among all 63 serum sequences was 0.056 (± 0.005), while among all 36 DBS sequences was 0.061 (± 0.005). Regarding the 36 paired serum and DBS samples, 30 pairs (83.3%) showed total identity (100% of similarity, genetic distance = 0.000), while three pairs of sequences (8.3%) showed 99.99% of similarity (genetic distance = 0.001) and 2 pairs of sequences (5.6%) showed 99.96% of similarity (genetic distance = 0.004). Only one pair of serum and DBS sequence (2.8%) presented less similarity (98%) with genetic distance = 0.02.

**Table 6:** HBV DNA sequencing in DBS samples correlated to HBV viral load, biochemical and serological markers.

Marker	DBS samples sequenced (n = 36)	DBS samples not sequenced (n = 27)	ρ
Mean serum viral load [log UI/mL (± SD)]	5.06 (± 2.22)	3.36 (± 1.26)	0.0003
HBeAg+ [n (%)]	14 (38.89)	04 (14.81)	0.04
Anti-HBe+ [n (%)]	21 (58.33)	24 (88.89)	0.01
Anti-HIV+ [n (%)]	04 (11.11)	00 (0.00)	0.12
ALT* mean [U/mL (± SD)]	17.00 (± 14.86)	12.12 (± 5.85)	0.07
AST* mean [U/mL (± SD)]	32.94 (± 36.65)	20.69 (± 12.73)	0.06
GGT *mean [U/mL (± SD)]	99.94 (± 156.79)	34.62 (± 32.32)	0.01
Alkaline fosfatase mean	79.74 (± 64.47)	54.83 (± 23.24)	0.06
Total bilirrubin mean	0.35 (± 0.39)	0.5 (± 0.84)	0.34
Indirect bilirrubine mean	0.27 (± 0.26)	0.39 (± 0.71)	0.35

*ALT alanine-aminotransferase, *AST* aspartate-aminotransferase, *GGT* gama-glutamyltransferase (GGT).

Phylogenetic analysis showed a higher prevalence of genotype A, followed by F, D and E (Fig. 2A). In serum samples, 41/63 = (65.08%) were classified as genotype A (subgenotypes A1: 36; A2: 5), followed by genotype F (17/63 = 27%; subgenotypes F2: 15; F4:2), genotype D (3/63 = 4.8%; subgenotypes D2: 1; D3: 1; D4: 1;) and E (2/63 = 3.2%). As these samples came from two different regions of Brazil (Northeast and Southeast), the frequency of each genotype by region was evaluated. It was observed a greater presence of genotype F in individuals from the Northeast (48.1%), while in Southeast, genotype A was more frequent (80.6%) (p = 0.0014).

Regarding DBS samples, there was 100% agreement with the genotype observed in the respective paired serum sample, as illustrated in the phylogenetic tree containing the 36 serum and DBS pairs (Fig. 2B). From them, 23 pairs belonged to genotype A (63.8%), 10 to genotype F (27.8%), 2 to genotype D (5.6%) and one to genotype E (2.8%). Only one pair was located in different branches in the phylogenetic tree, which may suggest the classification of these two samples in distinct subgenotypes.

In addition, variables as viral load, HBsAg titers, ALT and AST values, HBeAg, anti-HBe and anti-HIV positivity in relation to the different genotypes/subgenotypes and to the sequencing result were evaluated. Genotype A was statistically associated with low levels of AST and higher HBsAg titers when compared to genotype F (p < 0.005). Furthermore, sequenced samples had higher HBV viral load and HBeAg positivity than not sequenced samples (p < 0.05) while not sequenced samples had lower anti-HBe positivity than sequenced samples (p = 0.01).

Nucleotide sequences of serum and DBS were analyzed for HBsAg/polymerase mutations. All samples presented at least one polymorphism; most of them were neutral mutations. Overall, ~ 98% of polymorphisms observed in serum were also found in their paired DBS samples. The presence of HBV surface antigen-related mutations were identified in 10 serum samples (15.87%), with 144D → A (1), 133 M → I (1), 100Y → C (6), 109L → V (1) and 109L → R (1) being the most frequent mutations (Table 7). The paired DBS was available for 6 sequences and presented 100% concordance with the HBsAg mutations described in serum.

**Table 7:** Characteristics of samples with clinically-relevant mutations.

Sample	HBe/anti-HBe	Treatment	Genotype	Viral load*	HBsAg mutations	RT^Ɨ^ mutations
TSO1828	+/−	No	A1	7.8	V96A, M103I, T118A, K122R, F134L, **D144A**, V168A, V180A, S207T	I53L, V112I, N122H, Y126C, M129L, V142A, V163I, Q215H, I253V
TSO1836	−/+	No	A1	2.52	S34L, I68T, **M133I**, S193L, S204N, S207N	N122H, M129L, V142I, W153R, V163I, I253V
TSO1908	−/+	No	A1	3.74	**Y100C**, S207N	N122H, M129L, W153R, V163I, I253V, T259S, V278I
TSO1950	−/+	No	A1	3.55	S45A, L49P, S61L, **Y100C**, T189I, S207N	I53S, S109P, R110G, N122H, M129L, W153R, V163I, I253V, T259S, V278I
TSO2048	−/+	No	A1	3.16	P46HP, I81IT, **Y100C**, Y161F, S174NS, S193LS, Y206HY, S207N, P214LP	I103IV, N122H, M129L, W153R, V163I, V214AV, I253V, V266I, V278IV
TSO2069	+/−	No	A1	7.3	L49R, I68T, **Y100C**, Y161F, S207N, I208T, S210R	I53V, N122H, M129L, W153R, S159T, V163I, S219A, I253V, V278I, A307C
TSO2073	−/+	Yes (ETV)	A1	2.53	**L109V**, V184A, T189I, S204N, S207N, I208T	I53V, S117C, N122H, Q125K, M129L, V163I, I253V
LC40	+/−	No^#^	F4	5.14	**L110I, I195M**	T118N, H122Y, N123D, **L180M**, **M204V**, T237A, T259A, D271E
LC87	+/−	Yes (ETV)	A1	3.26	**Y100C**, A194V, S207N	N122H, M129L, W153R, V163I, I253V, V278I, F296I, A297PST
LC110	−/+	No	A1	3.27	G44E, S45A, L49P, **Y100C**, S204R, Y206V, S207N	A38AT, I53S, M129L, W153R, V163I, K212T, V214G, N238T, I253V, H271Q
LC111	−/+	No	F2	3.23	**L109R,** L26R, T27R, L109R, L127F	H35Q, N123D, M129L, S135F, S137T, N246S, T259S, V266I, D271E, T313A

*log UI/mL; ^Ɨ^RT: Reverse-transcriptase; ^#^HIV-treated patient. ETV: Entecavir. TSO: Samples from Southeast Brazil. LC: Samples from Northeast Brazil. Clinically relevant mutations are marked in bold.

Significant values are in bold.

One patient had the double resistance polymerase mutation rtM204V/I-rtL180M, in both serum and DBS. This is a 52-year-old man, heterosexual, from Northeast region, HIV co-infected, self-declared history of syphilis in the past, HBeAg + /anti-HBe-, subgenotype F4, HBV load in serum of 5.14 log UI/mL (5.67 copies/mL).

## Discussion

Broad access to HBV molecular diagnosis is a basic condition for starting therapy and monitoring the emergence of drug-resistant strains. However, ensuring this access is still a challenge in remote places and low-income settings^1,11,16^. In these cases, when the gold-standard tests are not available, the use of DBS and *in-house* techniques play a role in providing molecular diagnosis to hard-to-reach populations. Here, *in-house* qPCR was employed to detect and quantify HBV DNA in DBS samples. In addition, the applicability of DBS for sequencing purposes, as HBV genotyping and tracking mutations, was evaluate.

In this study, the detection limit of HBV in DBS by qPCR was 852.5 copies/mL (~ 250 IU/mL). Other studies using COBAS^®^ TaqMan^®^ HBV adapted for DBS have described detection limits of 914 IU/mL^19^ and 1400 IU/mL^24^. These discrepancies can be explained by differences between commercial and *in-house* methods, such as the target gene (*S/Pol* vs. *Pre-Core/Core* genes) and several automated steps.

The qPCR in DBS samples presented good agreement (86.40%), sensitivity (77.63%) and excellent specificity (100%) compared to serum samples. In-house qPCR was used as reference since this method was previously optimized and compared to commercial methods^25^ Some positive samples by commercial method were excluded to compare the results obtained in DBS what could impact in the estimation of sensitivity and specificity of the assay in this study and it is considered a limitation of the present work. Some studies carried out in DBS showed higher sensitivity values^26–29^, however most of them were carried out using commercial methods and with a smaller sample size. Moreover, satisfactory values of viral load and detection limit were observed in the repeatability and reproducibility analysis, demonstrating the reliability of the method.

Although HBV viral load was higher in serum compared to DBS, we observed good agreement between results from qPCR in serum and DBS as demonstrated by kappa value. Bland–Altman analysis demonstrated only few discordant results between qPCR in serum and DBS probably due to small differences in the performance of the assay among those samples. Other studies also demonstrated good correlation between qPCR for HBV in serum and DBS^19,26,27^. These results reinforce that the qPCR using DBS samples may be an economic and efficient alternative to HBV quantification in settings where serum sampling is not accessible.

Some variables were associated with qualitative and quantitative detection of HBV DNA in DBS, such as higher viral loads detected in qPCR, higher HBsAg values, HBeAg positivity and the absence of anti-HBe in the respective serum samples. Other studies have reported a greater HBV DNA detection in DBS samples with higher viral loads detected in serum^19,29,30^. Likewise, greater HBsAg and HBeAg detection, which in general are related to viral replication, were also associated with success in HBV detection by qPCR. In agreement, it was observed that anti-HBe reactive samples were more likely to have negative results in DBS, being related to a lower viral replication and, consequently, lower viral genome detection^31^.

Despite DBS is commonly used in mass serological screening, studies reporting its use for sequencing purpose are scarce and the robustness of DBS for molecular characterization is still an issue. Consequently, in low-income settings virus diversity is seldom determined, contributing to the lack of association of HBV DNA diversity and the hepatitis B phenotypes^32–34^.

In this study, we also investigated the genetic variability of HBV variants in 63 samples and evaluated the applicability of DBS for molecular epidemiology studies.

The comparison between paired serum and DBS sequences revealed great similarity, ranging from 98 to 100%. Phylogenetic analysis demonstrated that genotypes A and F were the most prevalent. Individuals infected with HBV/A showed higher values of HBsAg and lower AST titters compared to individuals infected with HBV/F (p < 0.005). Croagh et al.^35^ reported that HBV/A was associated to chronicity, while infection promoted by HBV/F tends to have limited progression and beneficial evolution, which may explain the difference in HBsAg levels.

Regarding subgenotypes, in both serum and DBS, HBV/A1 (57.1%) was the most prevalent, followed by HBV/F2 (23.8%), A2 (7.9%), F4 (3.2%), E (3.2%), D2, D3 and D4 (1.6% each). As have been stated, genotypes A, D and F are the most prevalent in Brazil^36–39^. On the other hand, genotype E is rarely detected in Brazil and when found, is often linked to the recent waves of African migration^38,39^. Here, genotype E was identified in two individuals from Angola living in Brazil. In agreement, phylogenetic analyses indicate that these HBV/E isolates are closely related with strains from Angola and Guinea.

It is noteworthy that HBVA1 was the most prevalent subgenotype in the Southeast, while F2 was the most frequent in Northeast. High proportion of HBV/F in Northeast was previously observed by Mello et al.^36^, and more recently, confirmed by Lampe et al.^38^. Phylogenetic analyses revealed that samples from the Northeast and Southeast had a heterogeneous distribution along the tree, suggesting that HBV strains circulating in these two regions don’t present a monophyletic origin. Brazil is a continental country where differences in colonization influenced the HBV genotype distribution along the regions^39^. However, expressive migration flows between the Northeast and Southeast over time may play a role in HBV dispersal and mixing of viral variants. Although this study does not suggest a distinction between the isolates from the two regions, studying the HBV variability distribution by region is crucial for increasing the knowledge of HBV dispersal patterns and for surveillance of viral variants circulating in chronic carriers.

In this study, 10 serum sequences displayed mutations in HBsAg gene. All mutations found in serum were also detected in the respective DBS sequences (when available), showing the potential of this sampling as an alternative for molecular analysis. All the amino acid changes were detected within the major hydrophilic region (MHR), where the determinant “a”, the main target of B and T cells, is located. Mutations in this region can affect the antigenicity of HBsAg and may be related to occult HBV infection and escape from vaccine-induced immunity^40^.

Overall, Y100C was the most frequent substitution, being present in 6/10 sequenced samples. Despite this mutation has been associated with occult HBV infection^41–43^, in this study all carriers were HBsAg-positive. As demonstrated by Mello et al.^44^, Y100C alone may not affect HBsAg production, secretion or HBsAg affinity by commercial serological assays, as for our samples. Thus, the presence of other potential reasons that may influence on HBsAg detection should be further investigated. In addition, L109R/V mutations were present in 2/10 samples and have been related to HBV vaccine escape and virus evasion to the host immune system^7,45^.

Regarding resistance mutations, the double rtM204V/I-rtL180M mutation in polymerase gene (to lamivudine, telbivudine and entecavir—partial) was observed in both serum and DBS samples from an HIV-treated patient. As observed by Komas et al.^18^, this finding reinforces the robustness of DBS to detect clinically relevant mutations. The accurate detection of mutations with clinical significance, as vaccine-escape and resistance mutations, is essential for better design immunization strategies and managing chronic carriers under antiviral therapy.

Our study has some limitations such as the selection of samples with viral loads above 1000 copies/mL. In addition, the assay described herein target only one region S/Pol of the HBV genome. Moreover, DBS is a less sensitive method providing a lower HBV quantification when compared to the gold-standard serum sampling, which can lead to an underestimation of viral loads. Due to this limitation, optimizations are still necessary to provide a larger number of sequenced DBS samples. DBS samples could be useful for initial screening followed by second test in serum samples before to initiate the treatment. In addition, DBS could be used to evaluate mutations and for molecular epidemiology studies when serum sampling is not available. At our knowledge, this study analysed the largest number of HBV sequences using DBS available in literature, thus contributing to increase the knowledge in this setting.

In conclusion, our results reinforce the applicability of DBS for molecular analyses, such as HBV quantification and sequencing, using *in-house* techniques. In low-income settings and remote locations, where the gold-standard tests are not available, the use of DBS for molecular purpose may be a convenient and economical alternative, increasing the access to HBV diagnosis. Providing alternative diagnosis methods to low-income and hard-to-reach population, who are frequently unaware of their carrier status, may close important gaps in HBV control.

## Material and methods

### Participants

A total of 141 individuals were included in this study: Group 1 was composed by 92 HBsAg and HBV DNA reactive individuals in serum with HBV viral load greater than 2.5 IU/mL by COBAS^®^TaqMan^®^ HBV (Roche Diagnostics, Branchburg, NJ, USA) or Abbott RealTime HBV (Abbott Diagnostics, Des Moines, USA). These subjects were recruited from ambulatories and laboratories related to the diagnosis and monitoring of viral hepatitis located in the states of Ceará and Rio de Janeiro of Brazil from 2011 to 2015. Group 2 (negative control) was composed by 49 healthy volunteers with no HBV or HCV serological markers (HBsAg, anti-HBc and anti-HCV) and with serum and DBS samples. These samples were collected in 2010 in hepatitis educational events and were part of the biorepository of the Laboratory of Viral Hepatitis of the Oswaldo Cruz Institute, with operation reviewed and approved by the institutional ethical committee on December 16, 2013. All individuals agreed to participate to the study by signing an informed consent. Sociodemographic and risk behaviour data were obtained through the application of a questionnaire. The study protocol was approved by the Research Ethics Committee of the Oswaldo Cruz Institute (number CAAE: 34055514.9.0000.5248), in accordance with the Declaration of Helsinki. Inclusion criteria were patients with more than 18 years old of any gender, race or ethnicity. Individuals with advanced cirrhosis (Child–Pugh B and C) and hepatocellular carcinoma were excluded from the study. All methods were performed in accordance with the relevant guidelines and regulations.

### DBS and serum samples

Blood samples were collected by venipuncture and provided the paired samples of serum and DBS. To prepare the DBS samples, seventy-five microliters of whole blood were distributed in Whatman 903 paper (GE Healthcare, Life Sciences, Little Chalfont, United Kingdom), according to manufacturer’s instructions. After blood inoculation, each DBS sample was dried at room temperature for at least 4 h and it was placed into zip-locked bag with silica gel desiccant sachet and stored at -20 °C.

### Biochemical and serological tests in serum

Serum levels of biochemical markers of liver function as alanine-aminotransferase (ALT), aspartate-aminotransferase (AST), gama-glutamyltransferase (GGT), direct and indirect bilirubin and alkaline phosphatase (AP) were determined using a photometric technique (ICMA) (Liason, Diasorin, Varceli, Italy).

Serum samples were used for detection of HBV, hepatitis C virus (HCV) and human immunodeficiency virus (HIV) serological markers with commercial tests. The detection of HBsAg (Biokit, Buenos Aires, Argentina), anti-HBc (Biokit, Buenos Aires, Argentina), anti-HBc IgM (Symbiosis Diagnóstica, Leme, Brazil), anti-HBs (Biokit, Buenos Aires, Argentina), anti-HIV (RPC Diagnostic Systems, Yablonevaya, Russia) and anti-HCV (Diasorin, São Paulo, Brazil) were performed using immunoenzymatic commercial methods, according to the instructions of each manufacturer. HBeAg (Roche Diagnostics, Rotkreuz ZG, Switzerland) and anti-HBe (Roche Diagnostics, Rotkreuz ZG, Switzerland) were performed in HBsAg positive samples.

### HBV DNA extraction in serum and DBS

HBV DNA was extracted from serum samples using a commercial kit (High Pure Viral Nucleic Acid Kit, Roche Diagnostics, Mannhein, Germany) following manufacturer's instructions. The same kit was used for HBV DNA extraction from DBS, according to Bezerra et al.^22^. Thus, three circles of 3 mm of DBS were directly used in DNA extraction and all the steps recommended by manufacturer's were carried out.

### HBV quantification

Serum samples were submitted to the commercial assay COBAS^®^ TaqMan HBV^®^ Test (Roche Diagnostics, Branchburg, NJ, USA) or to the commercial test Abbott Real Time HBV (Abbott Diagnostics, Des Moines, USA) for HBV-DNA quantification, according to the availability of the test and following the manufacturer’s instructions. All results were presented in copies/ml for comparative purposes. Therefore, the quantification values obtained by COBAS^®^ TaqMan^®^ HBV and Abbott RealTime HBV needed to be converted for copies/mL. For COBAS^®^ TaqMan^®^ HBV, the value was multiplied by 5.82 (conversion factor) to obtain the result in copies/mL, while for Abbott RealTime HBV the value in UI/mL was multiplied by 3.41.

Serum and DBS samples were also quantified using *in-house* real-time PCR (qPCR) as previously described^6^. For analysis of serum and DBS samples, the DNA was added to a PCR mix in a concentration of 25 ng/μL. All samples were tested in duplicate.

A serial dilution panel was used to assess analytical sensitivity and to determine the limit of detection of the qPCR in DBS samples. Dilution panel was made as previously described^22^. DBS samples ranging from 10^0^ to 10^7^ copies/mL, as estimated in commercial quantitative method, were tested in duplicate.

The repeatability of qPCR was evaluated by testing eight times in the same reaction a HBV DBS sample with high viral load (high positive control, HPC, 3.41 × 10^10^ copies/mL) and a HBV DBS sample with low viral load (low positive control, LPC, 3.41 × 10^4^ copies/mL). The reproducibility was obtained by testing HPC and LPC eight times in the same reaction, twice a day, for three days by three different operators^46^.

To demonstrate the precision of qPCR, a DBS sample containing 4log10copies of HBV/mL, previously determined by commercial quantitative method, was tested 20 times in the same reaction^46^.

### HBV DNA qualitative detection and S/Pol genes sequencing

HBV DNA from both serum and DBS samples were used for PCR amplification and sequencing of the overlapped small envelope and polymerase genes (S/Pol regions), producing a DNA fragment of ~ 900 bp, as previously described^23^.

The identity of the sequences was checked through the BLAST tool algorithm. Sequences representative of known genotypes and sub-genotypes were retrieved from GenBank and included in phylogenetic analysis. Phylogenetic trees were constructed with MEGA 7.0 software^47^ using the Maximum Likelihood method and General Time Reversible substitution model with gamma distribution, as the best-fit model.

To assess the presence of mutations in both viral envelope and in the reverse transcriptase domain of viral polymerase, the sequences obtained were submitted to Geno2pheno HBV algorithm (Max-Planck-Institut für Informatik, Germany, at http://hbv.geno2pheno.org/index.php). This algorithm also reports a trend in the sample's phenotypic resistance to five antiretroviral drugs.

### Data analysis

Descriptive statistical analysis was performed with calculation of means and standard deviation, with a preliminary assessment using contingency tables and respective statistics. Categorical variables were compared between groups using the chi-square test or Fisher’s exact test, and continuous variables were analyzed using the Mann–Whitney U test. A p-value of < 0.05 was considered significant.

To evaluate the effectiveness of the *in-house* qPCR for DBS samples, we measured precision, reproducibility, repeatability according to each reaction, per day, by operator and total. In addition, sensitivity and specificity were also determined.

Concordance between the results obtained for the DBS and sera samples was assessed using the Kappa index (k). According to international standards, findings should be interpreted as follows: < 0.20 corresponds to poor agreement; 0.21–0.40 as fair agreement; 0.41–0.60 as moderate agreement; 0.61–0.80 as good agreement, and 0.81–1.00 corresponds to very good agreement.

HBV DNA viral load correlation was calculated using Pearson correlation test. P value < 0.05 was considered significant.

Statistical analysis was determined using GraphPad InStat 3 (GraphPad InStat Software, San Diego, United States) and graphs were done using MedCalc Software v.9.6.4.0 (MedCalc Software, Belgium).

### Ethical approval

The study protocol was approved by the Research Ethics Committee of the Oswaldo Cruz Institute (number CAAE: 34055514.9.0000.5248).

## Data Availability

All relevant data is available in the manuscript.
